# Examination of the impact of the *Get SET Early* program on equitable access to care within the screen-evaluate-treat chain in toddlers with autism spectrum disorder

**DOI:** 10.1177/13623613221147416

**Published:** 2023-01-11

**Authors:** Christie Pham, Elizabeth C Bacon, Andrea Grzybowski, Cynthia Carter-Barnes, Steven Arias, Ronghui Xu, Linda Lopez, Eric Courchesne, Karen Pierce

**Affiliations:** University of California, San Diego, USA

**Keywords:** autism spectrum disorders, development, family functioning and support, health services, race and ethnicity

## Abstract

Delays in autism spectrum disorder identification/services could impact developmental outcomes. Although trends are encouraging, children from historically underrepresented minority backgrounds are often identified later and have reduced engagement in care. It is unclear if disparities exist throughout the screen-evaluate-treat chain, or if early detection programs such as *Get SET Early* that standardize these steps are effective countermeasures. Pediatricians/primary care providers administered Communication and Symbolic Behavior Scales IT Checklist screens at 12-, 18-, and 24-month well-baby examinations, and parents designated race, ethnicity, and developmental concerns. Toddlers who scored in the range of concern, or whose pediatricians/primary care providers had concerns, were referred for evaluations. Rates of screening and evaluation engagement within ethnic/racial groups were compared to U.S. Census proportions. Age at screen, evaluation, and treatment and quantity was compared across groups. Regressions examined whether key factors were associated with ethnicity or race. No differences were found for mean age of screen, evaluation, initiation of behavioral therapy, or quantity received between racial and ethnic groups. Historically underrepresented minority children were more likely to fall into the range of concern, referred for evaluations, and have their parents express developmental concerns. Although there remain gaps within the pipeline, implementation of systemized programs can be effective in ensuring equitable access to resources across communities.

**Lay abstract:**

Delays in autism spectrum disorder identification and access to care could impact developmental outcomes. Although trends are encouraging, children from historically underrepresented minority backgrounds are often identified at later ages and have reduced engagement in services. It is unclear if disparities exist all along the screen-evaluation-treatment chain, or if early detection programs such as *Get SET Early* that standardize, these steps are effective at ameliorating disparities. As part of the *Get SET Early* model, primary care providers administered a parent-report screen at well-baby examinations, and parents designated race, ethnicity, and developmental concerns. Toddlers who scored in the range of concern, or whose primary care provider had concerns, were referred for an evaluation. Rates of screening and evaluation engagement within ethnic/racial groups were compared to US Census data. Age at screen, evaluation, and treatment engagement and quantity was compared across groups. Statistical models examined whether key factors such as parent concern were associated with ethnicity or race. No differences were found in the mean age at the first screen, evaluation, or initiation or quantity of behavioral therapy between participants. However, children from historically underrepresented minority backgrounds were more likely to fall into the range of concern on the parent-report screen, their parents expressed developmental concerns more often, and pediatricians were more likely to refer for an evaluation than their White/Not Hispanic counterparts. Overall results suggest that models that support transparent tracking of steps in the screen-evaluation-treatment chain and service referral pipelines may be an effective strategy for ensuring equitable access to care for all children.

## Introduction

The human brain undergoes an unparalleled period of growth and development between birth and 3 years of age as synaptic density in the prefrontal cortex, the brain region centrally involved in higher-order social behavior, doubles (Huttenlocher, 1979) and brain size reaches 80% of its adult volume (Gilmore et al., 2018). There is evidence suggesting that children with autism spectrum disorder (ASD) who begin their treatment within this period of high plasticity are at an advantage relative to children who start at a later age (Gabbay-Dizdar et al., 2021). Thus, understanding factors that impact the timing of treatment onset for autistic children is of highest public health importance.

Although trends are encouraging (Maenner et al., 2021), in the past, autistic children from historically underrepresented minority (HURM) backgrounds are often identified with the disorder less frequently (Baio, 2014; Baio et al., 2018) and at older ages (Mandell et al., 2009; Zuckerman et al., 2014), use fewer services (Zuckerman et al., 2017), and are more likely to have unmet service needs (Iland et al., 2012; Zuckerman et al., 2017) than their White counterparts. For example, Mandell et al. (2009) previously found that historically minoritized children were 1.47 to 2.63 times less likely to have documentation of ASD in their health care or education records despite meeting criteria for the disorder, and Black children received ASD diagnoses 1.6 years later than their White counterparts (Mandell et al., 2002). More recently, Baio et al. (2018) estimated ASD prevalence 7% and 22% greater among White children than Black and Hispanic children, respectively. Factors that may influence disparities include affordability and challenges in finding an “effective” conventional healthcare provider (Lindly et al., 2018) as well as levels of autism awareness, parental education, and resources availability in individual communities (Colbert et al., 2016; Durkin et al., 2010; Fountain et al., 2011; Khowaja et al., 2015; Liptak et al., 2008; Pedersen et al., 2012; Zuckerman et al., 2013) possibly stemming, in part, from structural racism (Broder-Fingert et al., 2020).

Although limited, data suggest that universal screening can positively impact standards of care within varying communities (Council on Children with Disabilities et al., 2006; Landa et al., 2011). Tools such as the Parents’ Evaluation of Developmental Status (PEDS; Glascoe, 2010), the Modified Checklist for Autism in Toddlers-Revised with Follow-Up (M-CHAT-R/F; Robins et al., 2014), and the Communication and Symbolic Behavior Scales IT Checklist (CSBS; Wetherby & Prizant, 2002) meet many of the criteria set forth by the American Academy of Pediatrics (AAP; American Academy of Pediatrics, 2018a) and are commonly used in practice. Although these screening tools vary in their levels of sensitivity and positive predictive value (e.g. Guthrie et al., 2019), they have shown considerable efficacy in allowing primary care providers to identify ASD (Centers for Disease Control and Prevention, 2018; Glascoe et al., 2007), lowering the mean age of first diagnosis (American Academy of Pediatrics, 2018b; Pierce et al., 2011, 2021; Robins et al., 2014; Wetherby et al., 2008). The AAP recommends general developmental screening at 9, 18, and 30 months and autism-specific screening at 18- and 24-month well-baby visits. In their 2020 report (Hyman et al., 2020), the AAP holds their stance on these developmental surveillance timepoints due to the prevalence of ASD and the potential for evidence-based interventions in improving outcomes. Thus, the use of standardized screening tools seems to be an important first step toward building a pathway from screening to evaluation to treatment. For example, in cases where clinicians have used standardized screening tools, referral rates for evaluations have increased for families within lower socioeconomic levels (Halla et al., 2016; Pierce et al., 2021), pediatricians served a higher proportion of Medicaid clients (i.e. those potentially in increased financial need; Sand et al., 2005), and pediatricians were more likely to report a >10% rate of developmental problems (Sand et al., 2005). In addition, when these tools were used, referral rates at younger ages, such as the 12-month age point, increased compared to the number of referrals provided during the previous year (Hix-Small et al., 2007). In one study, standardized screening reduced the wait duration for the identification of a developmental delay by between 59% and 68%, and the wait duration for referrals also decreased by 64%–70% (Guevara et al., 2013). With the implementation of these tools and systematic support, parents have reported increased satisfaction with overall care and physicians have endorsed their efficacy in aiding identification when the screens are incorporated into the office flow (Shannon & Anderson, 2008). Moreover, studies in which screening tools are utilized report ethnic and racial participation rates that are comparable to the population’s demographic ratios (Goin-Kochel et al., 2006; Zuckerman et al., 2021).

Despite improvements and the support for standardized screening (Barton et al., 2012; Frankenburg, 2002; Hyman et al., 2020; Radecki et al., 2011), specific data regarding standardized screening performance across different ethnic and racial groups are somewhat limited in scope. Although many studies include relatively large samples from Hispanic or minority backgrounds (e.g. Hirai et al., 2018), the focus of such studies is often to examine the administration of screening tools. Windham et al. (2014) examined scores of different ethnic groups (81.1% Hispanic) on the Modified Checklist for Autism in Toddlers (M-CHAT; Robins et al., 2001) and the Ages and Stages Questionnaire (ASQ; Squires et al., 1997); however, the researchers analyzed patient performances based on a nonstandardized Spanish version of the M-CHAT for 62.5% of their sample and did not use the follow-up interview that significantly contributes to the accuracy of the screener (Robins et al., 2014). Overall, while there are several studies examining the initial step of screening, apart from one study (Zuckerman et al., 2021), few studies examine the downstream, complex journey autistic children and their families face to move beyond screening and into engagement in evaluation and treatment services.

The aim of the present study was to determine if engagement in *Get SET Early* (Pierce et al., 2011, 2021), an early screening, evaluation, and treatment referral program, results in equal levels of engagement and performance between racial and ethnic groups along all levels of the screen-evaluate-treat chain. To do so, uptake rates within key metrics of the model were examined and stratified by ethnicity and race, including average screen age, screen range of concern rates, parent concern rates, pediatrician/primary care provider referral rates, average evaluation age, average treatment start age, and average hours of support services received.

## Methods

Data from the inaugural study on the *Get SET Early* model (Pierce et al., 2021) were leveraged here to examine differences in engagement in the screen-evaluate-treat chain between ethnic and racial groups. The *Get SET Early* program focuses on a three-step process, where S = Screen, E = Evaluate, and T = Treat, linking the critical elements of screening, evaluation, and treatment in a rapid fashion. The overall goal of the model is to detect as many cases as possible with ASD within a pediatric office by the second birthday. To support the model, a local pediatrician network of 203 pediatricians/primary care providers across 37 offices was established and pediatricians/primary care providers trained to administer a parent report broadband screening tool either on paper or digitally, at well-baby visits. While the overwhelming majority of providers in our network were pediatricians, a small minority were nurse practitioners or family medicine physicians. Thus, moving forward, the term “primary care provider” (PCP) will be used in reference to the provider who administered the screen. The pediatric offices within the network spanned from northern San Diego County to near the border of Mexico and consisted of a mix of community health centers, not-for-profit regional health care groups, and private practices. Several pediatric offices near the border of Mexico and in the southern region of San Diego County were deliberately recruited to participate to ensure that the program provided access to care for the Hispanic populations in these areas. Each office was offered the opportunity to use either paper or digital screeners. Decisions were made at the office level by office leadership and were based on the consideration of ease of integration into the current workflow. If a practice chose to use iPads, they were supplied the devices by the University of California San Diego (UCSD) Autism Center of Excellence (ACE) and did not need to purchase the iPads themselves. These iPads were also maintained by ACE staff. A brief review of key steps in the *Get SET Early* model and in the screen-evaluate-treat chain is as follows.

### Overview: SCREEN

**Figure 1.**
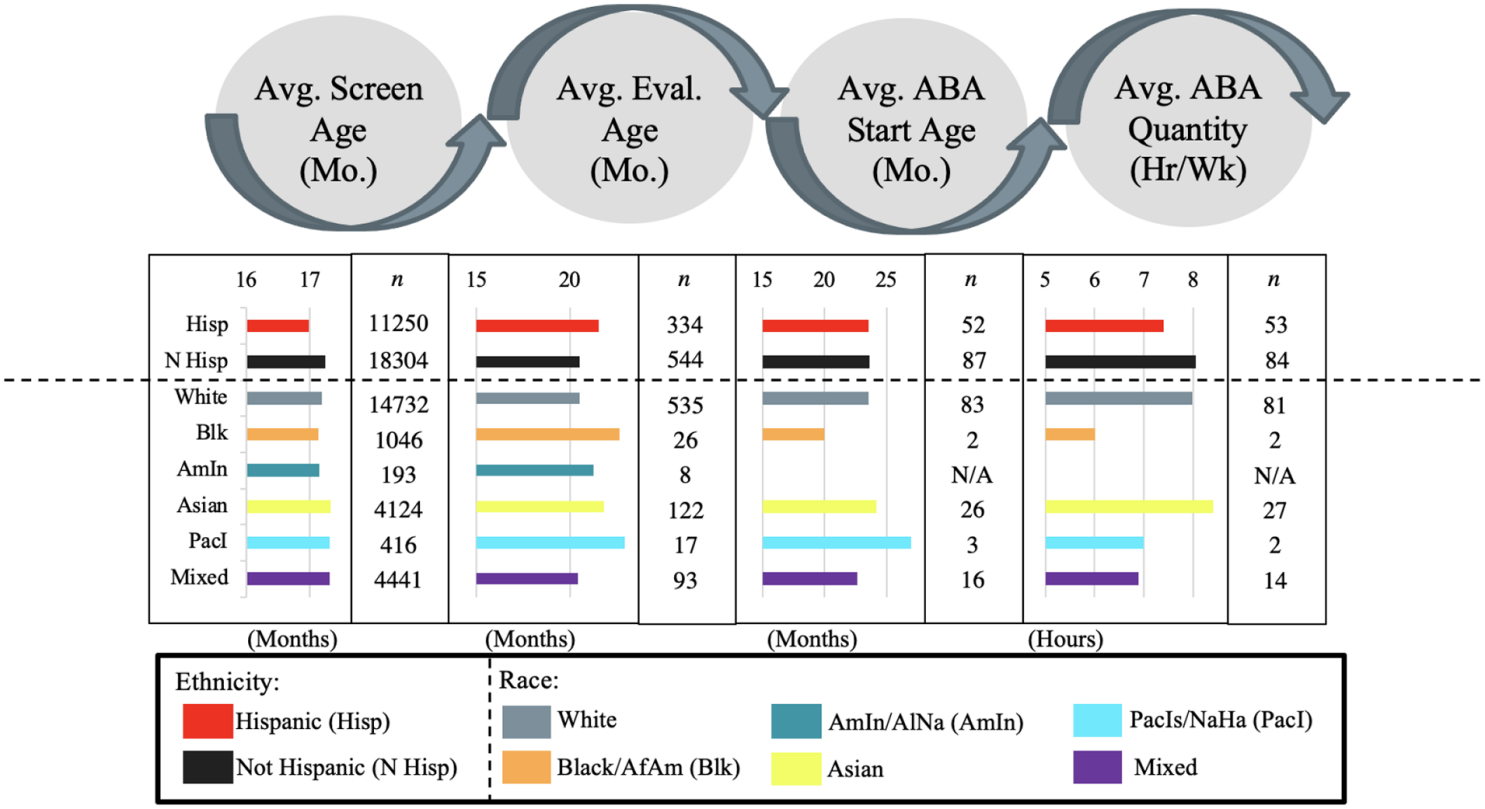
*Get SET Early* ages + ABA per week. Average screen, evaluation, and ABA-based therapy start age are portrayed for the different ethnic and racial groups. The average number of hours of ABA-based therapy per week for the most recent 6 months of treatment is also presented. The American Indian/Alaska Native group did not have reported ABA-based therapy statistics and thus are not included on these graphs. ABA numbers are different across start age and quantity as some parents, in their parent-report data, did not put a start date or list hours after they had indicated a start date.

The CSBS IT Checklist (Wetherby & Prizant, 2002) was chosen as the screening tool for the *Get SET Early* program due to its many strengths including its broadband nature (Wetherby et al., 2004, 2008); age-specific scoring from 6 to 24 months, administration time of about 5 min, and ease of re-administration at different ages (Pierce et al., 2021). In clinical practice, it detects toddlers with a range of developmental issues including language delay, global developmental delay, and ASD. Although the screen has limitations as a broadband screener in that it does not assess motor skills, these skills are typically assessed during well-baby visits by the PCP in accordance with AAP guidelines (American Academy of Pediatrics, 2022) and are further examined during the developmental evaluation should the toddler be referred. The screen consists of 24 questions that probe key domains of development including social communication, expressive speech, and symbolic behavior. The final question states “*Do you have any concerns about your child’s development?*” (Yes/No). Parents/caregivers answer the concern checkbox before the screen is scored, allowing them to indicate their concern on the screen prior to receiving the screen’s outcome. A toddler can fall into the range of concern within the social communication subdomain, symbolic behavior subdomain, or overall total score by scoring below the point threshold determined by their age. A total of 27,049 CSBS screens were completed on paper, and 30,554 were completed digitally using an iPad (29,330 English screens (95.99%) and 1224 Spanish screens (4.01%)). The demographic makeup of those who completed the digital screens and included race and/or ethnicity data was compared to those who completed the paper screens and included race/ethnicity data. No significant differences were found across these groups (see Supplemental Table 1). Therefore, because digital data allow for more accurate tracking than paper (e.g. number of screens administered), and eliminate adding errors associated with screens that require manual summations, only digital screening data were used (hereafter, eCSBS) to evaluate the screening portion of the study. Within the 30,554 digital screens, 29,554 (96.73%) toddler screens were utilized for the ethnicity comparisons and 24,952 (81.67%) were utilized for the race comparisons after removal of screens in which parents opted not to provide either ethnicity or race (for breakdown see Figure 1). Ethnicity and race analyses were performed independently of each other. Parents/caregivers were given the option to designate ethnicity, race, a combination of both, or neither based on their own identification. Thus, when analyzing toddlers from the Hispanic ethnic group, all associated races were included (e.g. White, Black/African American, American Indian/Alaska Native, Asian, Pacific Islander/Native Hawaiian, Mixed race, or had no race indicated); and when analyzing racial categories, all ethnicities were included (e.g. Not Hispanic, Hispanic, and decline to state).

There were no exclusionary criteria in the study; all toddlers received the screen when they received their well-baby visit. Specifically, caregivers completed the eCSBS in either English or Spanish during 12-, 18-, and 24-month well-baby check-ups on an iPad. Results of the screen were displayed on the iPad for review by the PCP prior to the well-baby visit and indicated the overall status of each of the domain areas of the eCSBS (concern/no concern), as well as the number of points a toddler scored either above or below the age cutoffs published by the screen developer in each domain. Using the iPad, PCPs designated whether they referred a child with a screen within the range of concern to UCSD for an evaluation. PCPs were under no obligation to refer to UCSD for an evaluation. On the digital versions of the forms, providers were able to indicate if they chose not to refer a child to UCSD, and why they made that choice. The multiple-choice answers available included no concern, referred elsewhere, patient not interested, reassess later, and others. Further details regarding this process can be found in the *2021 Get SET Early* manuscript (Pierce et al., 2021).

#### Outcomes related to screening phase

Number of screens administered, age at screen, parent concern rate, screen range of concern rate based on overall total score as well as stratified by subdomain, degree the screen fell into the range of concern by based on the number of points that exceeded the screen cutoff score for a given age, and evaluation referral rate.

### Overview: EVALUATE

Based on the CSBS screen and their own clinical judgment, PCPs referred a subset of toddlers who fell into the range of concern on the CSBS screen for an evaluation. Although toddlers who fell into the range of concern on the screen were not required to go to UCSD to receive their evaluation, families were informed that the evaluation at UCSD is free which may have incentivized choosing ACE over other evaluation centers that charge for their services. Furthermore, UCSD staff, including psychologists, were recruited and hired to communicate and give evaluations in Spanish, which may have encouraged Spanish-speaking families to utilize ACE for their evaluation. Toddlers received developmental evaluations by licensed clinical psychologists that included the Autism Diagnostic Observation Schedule (ADOS; Lord et al., 2001, 2012), the Mullen Scales of Early Learning (MSEL; Mullen, 1995), and the Vineland Adaptive Behavior Scales (VABS; Sparrow et al., 2005, 2016). At the conclusion of testing, psychologists entered one of the following diagnoses into a database based on assessment results and clinical judgment: ASD, ASD Features, Developmental Delay, Language Delay (LD), Other Issue, Previous LD, Typically Developing, or Typical Sibling of an ASD proband (for further details on diagnostic criteria see Pierce et al., 2021). Eight hundred ninety-seven toddlers aged 12–36 months completed one or more diagnostic evaluations (403 toddlers with ASD, 47 with ASD Features, 302 with other delays, and 145 CSBS false positives; for overall ethnic/racial breakdown, see Figure 1). If a toddler was evaluated at multiple ages, the toddler’s most recent diagnosis was used for all analyses. Thus, diagnostic categories included in the analyses are mutually exclusive. Testing results were reviewed with parents at the conclusion of testing and referrals for services were made as appropriate. Results were also summarized in a written report and mailed to the parent and referring PCP within a 2-week period.

#### Outcomes related to evaluation phase

Number of evaluations, age at evaluation.

### Overview: TREAT

Only toddlers who received an ASD diagnosis were included in the treatment engagement portion of the study to focus on how implementation of the screen-evaluate-treat chain impacts access to services for those with autism. Based on the ACE protocol, all toddlers were provided with a referral to the San Diego Regional Center (SDRC) for the appropriate treatment services unless they were already receiving them, and parents could choose to go to other providers outside of the Regional Center if they wished. As UCSD ACE does not currently provide therapy services, it was not available as a treatment provider option following diagnosis. Following appropriate parent consent, treatment engagement data were retrieved directly from SDRC, which leverages Part C funding (federal funding issued to California) in support of services for autistic children. SDRC treatment data consisted of authorization records and treatment plans that included the number of total service hours over a specific period of time, the name of the service provider, and, in most cases, the type of service. If the type of service was not indicated, data were matched across the authorization records and the treatment plan or confirmed with SDRC clinical staff. Parent-reported treatment data were also compiled to supplement gaps in treatment for subjects and included the start and end dates of treatment, the type of service, and the name of the service provider. Treatment engagement data were available for 301 toddlers out of the 403 with ASD (SDRC: 170, Parent-report: 131). However, as not all parents indicated both start age/date or the number of hours their child received, the number of subjects in each analysis was less than the overall number of entries (for breakdown see Figure 1). Start ages and quantity of Applied Behavioral Analysis (ABA)-based therapy received was utilized for treatment analyses as ABA-based therapy is a common and widely provided service provided following a diagnosis of autism.

It is important to note that although 100% of toddlers who were diagnosed with ASD were referred for treatment services based on ACE policy, treatment data were unobtainable if parents did not sign the parent consent form for a release of information. Furthermore, the number of treatment records obtained may be impacted by families moving, receiving services outside of ACE’s network, or parents not returning for follow-up visits after their initial evaluation. Treatment data were only collected after the initial evaluation is completed and treatment has begun. Thus, should families not return for further evaluations, their treatment data cannot be retrieved.

#### Outcomes related to treatment phase

Age at ABA-based therapy start, total amount of ABA-based therapy received, and average ABA-based therapy quantity per week averaged across the latest 6 months of treatment.

### Statistical analyses

Rates of screening and evaluation participation within ethnic and racial groups were compared to expected proportions based on San Diego County census data (U.S. Census Bureau, 2021) using binomial tests to assess for levels of community engagement. Regression models (logistic, linear) were used to examine if race or ethnicity contributed to expressed parent concern, screen falling into the range of concern, PCP referral, age at first evaluation, age ABA-based therapy was initiated, the total amount of ABA-based therapy received, and the average amount of ABA-based therapy received while accounting for variables such as child sex and age as appropriate. Analyses of variance (ANOVAs) were used to examine differences in performance across groups in screens that fell into the range of concern across subsections and ABA-based therapy engagement rates in the child’s latest 6 months of treatment. Subjects with no reported ethnicity or race were excluded from respective analyses.

Overall, the reported study included community involvement from PCPs and San Diego Regional Center providers who assisted in the implementation of the study and UCSD psychologists who were involved in the implementation of the study and interpretation and dissemination of the findings.

## Results

### Screen

**Figure 2.**
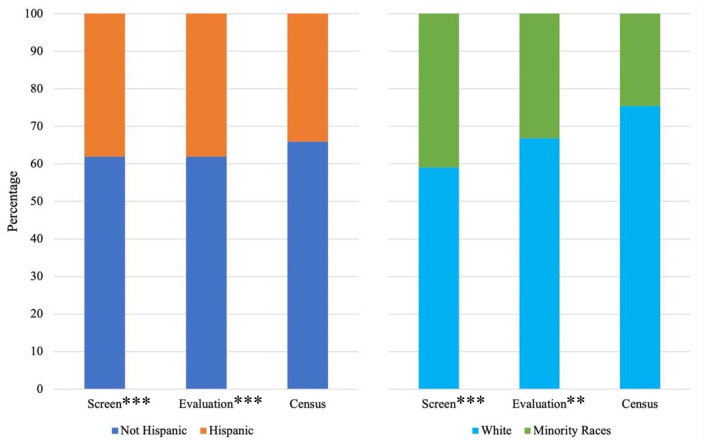
*Get SET Early* engagement levels versus U.S. Census ethnicity/race ratios. Ethnic and racial engagement at the screen and evaluation level in comparison with 2021 U.S. Census rates for San Diego County. As illustrated, the proportion of children from HURM backgrounds screened and evaluated occurred at slightly higher rates than expected based on Census data. **p < 0.01, ***p < 0.001.

**Figure 3.**
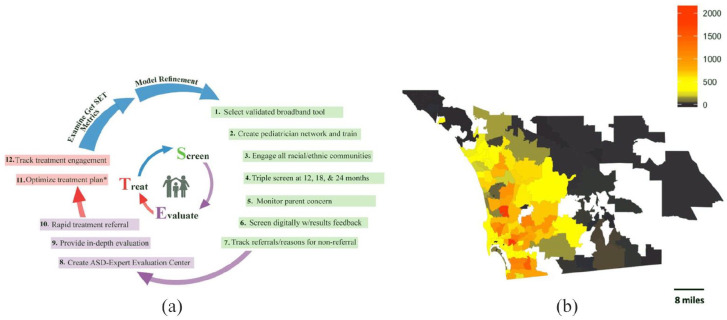
*Get SET Early* model and geographic distribution of screen region. (a) Schematic of the main tenets of the *Get SET Early* model, where S = Screen; E = Evaluate; and T = Treat (adapted from Pierce et al., 2021). (b). Geographic Distribution of *Get SET Early* Catchment Area. The colors in the figure represent the density of screening throughout San Diego County.

San Diego County is ethnically diverse with 34.1% of families reporting Hispanic or Latino ethnic background. Racially, 75.4% of individuals are White, 5.5% of individuals are Black, 1.3% are American Indian or Alaska Native, 12.6% are Asian, 0.6% are Pacific Islander or Native Hawaiian, and 4.6% are two or more races (Mixed). At the screen level, the *Get SET Early* program reached a higher number of Hispanic (*p* < .001) and minority race toddlers (*p* < 0.001) than anticipated based on percentages for those groups in San Diego County (U.S. Census Bureau, 2021; Figure 2), suggesting strong representation of those groups in our catchment area (Figure 3(b)). Overall, there were no differences in the average age of screen between ethnic and racial groups (i.e. ~17 months for all groups; *p* > 0.05; Figure 1).

**Figure 4.**
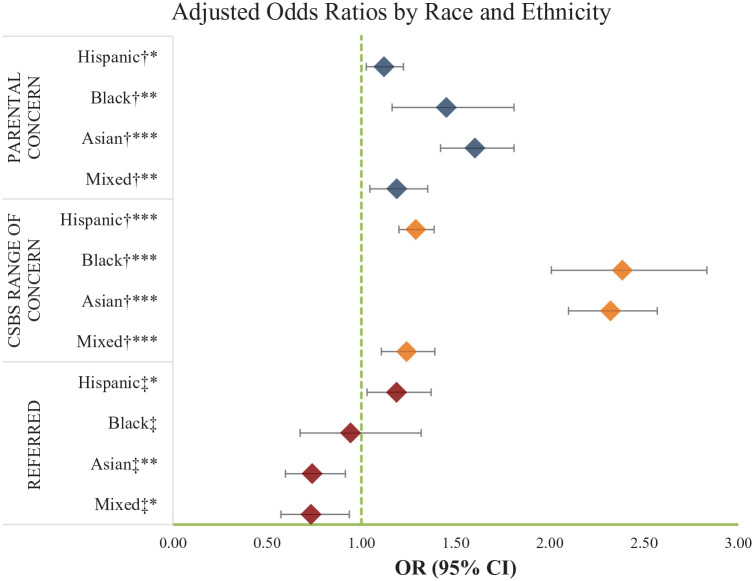
Logistic regression odds ratio plot. Odds ratio plots with 95% confidence intervals illustrating the impact of ethnicity and race on parental concern, eCSBS screen range of concern, and whether a toddler was referred for an evaluation. Reference Groups were as follows: Ethnicity: Not Hispanic; Race: White; Sex: Male; Concern: No concern. † = OR adjusted for toddler sex and age at first screening; ‡ = OR adjusted for toddler sex, age at first screening, and parental concern. Results indicated increased levels of parent concern, eCSBS range of concern, and PCP referral rates among Hispanic toddlers, and some elevated rates in various racial groups. *Note.* Subjects with no reported race were excluded from all race-based analyses. Subjects with no reported ethnicity were excluded from all ethnicity-based analyses. ◆= Adjusted *p < 0.05, **p < 0.01, ***p < 0.001.

Parents of Hispanic toddlers were significantly more likely to express concern about their child’s development on the CSBS screening tool than Not Hispanic parents, independently and when controlling for sex and screen age (Figure 4). Black/African American, Asian, and parents of Mixed race children were also significantly more likely to express concern than White parents (Figure 4).

Hispanic toddlers were also significantly more likely to fall into the range of concern for the screening tool than their Not Hispanic counterparts, independently (Figure 4) and when controlling for sex and screen age (Figure 4). Black/African American, Asian, Pacific Islander/Native Hawaiian, and Mixed race toddlers were also significantly more likely to fall into the range of concern for the screener than their White counterparts (Figure 4). A closer examination of screen performance revealed differences between the Hispanic and Not Hispanic groups in the number of age-specific points that the screen was in the range of concern within the Social subdomain score, *F*(1, 1435) = 11.32, *p* = 0.001 and the Symbolic subdomain score, *F*(1, 2855) = 17.64, *p* < 0.001, as well as the overall Total score, *F*(1, 1955) = 20.33, *p* < 0.001. Although there were no significant effects in the Social subdomain and Total score, there was a significant effect of race on the average number of points. The Symbolic subdomain score was in the range of concern by, *F*(5, 2181) = 6.51, *p* < 0.001. Follow-up comparisons using Tukey’s HSD (honestly significant difference) test revealed that the Black/African American (*M* = −1.34, *SD* = 1.62), American Indian/Alaska Native (*M* = −2.06, *SD* = 2.21), and Asian (*M* = −1.29, *SD* = 1.54) groups fell into the range of concern for the Symbolic subdomain by significantly more points than did the White group (*M* = −0.96, *SD* = 1.38).

### Evaluate

Hispanic toddlers were more likely to receive a referral for a developmental evaluation than their Not Hispanic counterparts, independently and when controlling for toddler sex and parent concern (Figure 4). The same pattern was observed for American Indian/Alaska Native toddlers who were referred significantly more than their White counterparts, but the opposite was seen for Asian and Mixed toddlers who were referred significantly less than their White counterparts (Figure 4).

Although evaluation demographics (for breakdown see Figure 1) were also generally consistent with San Diego demographics (U.S. Census Bureau, 2021), single-sample binomial tests similarly revealed that a higher number of Hispanic (*p* = 0.008) and minority race members (*p* < 0.001) followed through with the evaluation referral and were evaluated at our Center by a licensed psychologist (Figure 2).

Independently and when controlling for sex and screen age, there were no significant differences in the age toddlers came in for their intake evaluation between any of the groups (Figure 4).

### Treat

Of the ASD toddlers whose treatment records were reported, 59.31% of toddlers whose parents’ reported ethnicity and 60.77% of toddlers whose parents’ reported race had ABA records. At the treatment level, there was no significant difference in the age toddlers began receiving ABA-based therapy (Figure 1) across ethnic or racial groups. In both the overall amount of ABA-based therapy received and the average amount of ABA-based therapy received in each subject’s latest 6 months of treatment, there was no significant level of effect across ethnic and racial groups independently and when controlling for ABA-based therapy start age (Figure 1; *p* > 0.05 for all models). One-way between-groups ANOVAs for the average amount of ABA-based therapy received in the latest 6 months of treatment demonstrated that this pattern persisted when minority racial groups were considered separately or collapsed into a singular “Minority” group, *F*(5, 121) = 1.41, *p* = 0.23 and *F*(1, 125) = 0.36, *p* = 0.55, respectively.

## Discussion

Toddlers in San Diego County that participated in the *GET SET Early* program had almost identical mean age of screen (17 months), first evaluation (20 months), and ABA-based therapy start (23 months) regardless of their ethnic or racial background. This is an essential finding given that the infant and toddler brain is undergoing high rates of expansion and connectivity during this time period (Gilmore et al., 2018), and treatment delays of even a few months could adversely impact a child’s outcome (Gabbay-Dizdar et al., 2021).

Interestingly, the *Get SET Early* program reached significantly more children from Hispanic background than would be expected based on U.S. Census data. *The Lancet* commission on the future of care and clinical research on autism (Lord et al., 2022) notes that governments and service providers should monitor access to resource provision to ensure that HURM groups have access to appropriate services. There are two key tenets within the model that may have contributed to the success of this aspect of the program. First, a major tenet of the *Get SET Early* program is to actively seek out all communities (Figure 3(a), Step 3). This initial step was highly successful and pediatric offices spanning a range of demographic regions, including those near the border of Mexico, were readily inducted into the program. Second, another tenet is to screen digitally and track referrals to monitor service engagement across all groups (Figure 3(a), Steps 6 and 7). The mere act of transparent tracking using an iPad may have established checkpoints that supported equitable care. The concept of symptom monitoring supporting behavioral change/positive care has been observed in other health areas such as the field of diabetes (Ellis et al., 2007) and cancer identification (Marmot et al., 2013). Overall, active efforts to recruit several pediatric offices near the border of Mexico and within the southern region of San Diego County, lack of exclusionary criteria, opportunity to use the Spanish version of the screen, and Spanish-speaking clinical coordinators and psychologists at ACE may have also encouraged the engagement of Hispanic families.

During the first step, screen, the *Get SET Early* program utilizes a broadband tool, the CSBS IT Checklist (Wetherby & Prizant, 2002) to screen toddlers at well-baby visits for symptoms consistent with social communication delay. Initial symptom presentations for ASD may be subtle, thus, utilizing a broadband tool allows the program to pinpoint toddlers who might not be easily flagged for an increased likelihood of ASD, particularly in HURM groups that have been historically overlooked during screening (Baio, 2014; Baio et al., 2018) and diagnosis (Mandell et al., 2009; Zuckerman et al., 2014). Given that the screen is incorporated into well-baby visits, the *Get SET Early* program may lessen disparities in access to care by bringing developmental concerns to the forefront of a routine pediatric visit. This may assist parents who are unable to commit additional time, effort, or cost of setting up an ASD-specific visit with their PCP. Several reasons may explain why the number of Spanish screens was low in comparison to the English screens. First, offices were always offered both English and Spanish screens by the ACE team when the clinical liaison arrived at the office to collect completed forms. For the digital screens, both English and Spanish versions of the screen were available on the iPad at all times. However, it is up to the office front desk staff as to how many English versus Spanish screens they requested. Furthermore, there is considerably high staff turnover in medical offices, and while we were diligent with continued engagement with our pediatric network, it is possible that some front desk staff were more consistently offering both Spanish and English options than others. Second, the PCP for the child may not have been bilingual in Spanish. Thus, they may have offered the English screen to families who are bilingual so that the provider could answer the family’s questions about screen items. There is also a historical precedence of using English forms in medical practices in the United States, and this may have biased both the healthcare provider in which screen they offered as well as which screen version the family chose to fill out. In the future, adding a question regarding primary language spoken at home may better track the compliance of offices in offering both versions of the screen.

Interestingly, parents from Hispanic, Black/African American, Asian, and Mixed groups expressed significantly greater levels of concern compared to their Not Hispanic and White counterparts. While there are several possible interpretations for this, one may relate to the fact that minority groups may feel the need to assimilate to the mainstream culture or experience acculturation. These experiences may increase concerns surrounding their children and if they are developing to the standards of the foreign culture. This aligns with previous research examining the relationship between immigrant Hispanic populations’ acculturation profiles and parenting stress (Williams et al., 2017). Williams et al. (2017) found that Hispanic parents who were categorized as experiencing more stress from acculturation also reported less positive parenting behaviors as well as an increase in family conflict. This may suggest that parents who experience stress from acculturation may also view their children under a negative lens due to family conflict. Another possibility includes the idea that some parents may simply be advocating more and gaining medical providers attention for their children (e.g. due to past negative experiences or difficulties regarding medical care). Alternatively, this finding may highlight cultural differences in expectations for a child (Wang, 1993) or the level of developmental challenges of the children who were included and are worthy of future research. Although the American Indian/Alaska Native and Pacific Islander/Native Hawaiian group did not show significant differences in parent concern compared with the White group, this may be due to the smaller sample size for these groups which is consistent with, and a limitation related to, San Diego County demographics (U.S. Census Bureau, 2021).

Possibly related to increased rates of parental concern, toddlers from Hispanic, Black/African American, Asian, Pacific Islander, and Mixed toddlers fell into the range of concern for the CSBS screen at higher rates than their Not Hispanic and White counterparts. This may be related to the validity of the screen within HURM groups. Although performance within different ethnic and racial groups was examined in the initial validation of the screen (Wetherby et al., 2002), the initial validation study for the screen as noted in the CSBS DP Manual only included 120 Hispanic toddlers (5.5% of their total sample), 483 Black toddlers (23% of their total sample), 61 Asian toddlers (2.9% of the total sample), and 56 “Other” toddlers (2.7% of the total sample) (Wetherby et al., 2002). Although the authors state that these ratios were comparable to 2000 Census data on a race level and below 2000 Census data on an ethnicity level, the current study’s large sample size (i.e. ~14 times larger than the screen’s validation sample size) and overall higher number of HURM subjects reveal that future research should pay particular attention to the validity of the screen for these groups. In particular, researchers may want to examine if cultural differences influence the applicability of specific items on the screen (e.g. cultural normativity of playing with blocks) and adapt these items to better fit each group. However, the increased range of concern rates across HURM groups can be considered a strength of the *Get SET Early* program in its ability to highlight the need of these groups in receiving early access to developmental care. The higher screen range of concern rate within HURM backgrounds supports the idea that the previous rates of lower ASD identification within these groups (Baio, 2014; Baio et al., 2018) may be due to disparities within the healthcare system, and not differences in the prevalence of ASD within these groups. This is consistent with the Centers for Disease Control and Prevention’s (CDC) most recent prevalence report that noted, for the first time, a lack of significant differences in ASD prevalence between racial and ethnic groups (Maenner et al., 2021).

Although ethnic and racial factors did significantly increase the probability that a PCP would refer a toddler for an evaluation (e.g. Hispanic, Black/African American), parent concern remained a stronger predictor of PCP referral. This finding supports the implementation of screening during routine pediatric visits. Screening enables PCPs to recognize the need for developmental attention for HURM groups and may ameliorate disparities in access to care. Furthermore, the screen’s parent concern checkbox provides HURM parents with a platform and basis to concretize their worries. In contrast to toddlers in the Hispanic and Black groups, Asian and Mixed groups had significantly less referrals than their White counterparts. In other healthcare fields, the lack of trust in providers is a large barrier to accessing care for South Asians (for review, see Batra et al., 2019). Asian Americans have also reported significantly more risky behaviors in preventive care (e.g. not getting physical/dental exams annually, not consulting with a physician when experiencing unfamiliar physical symptoms; Courtenay et al., 2002) than European Americans, Hispanics, African Americans, Mixed Americans, and “Other” Americans (Courtenay et al., 2002). Therefore, it may be that the Asian group prefers to use the “wait and see” approach when it comes to developmental concerns and should be further studied in future research. Multiracial identity may also influence health care status (e.g. Ayers et al., 2010). Thus, impacts of an increased complexity in racial identification and development for mixed families/children should be examined in future studies to better elucidate the relationship between these groups and their interaction with the field of health care.

Although at face value the *Get SET Early* program appeared successful at ensuring equitable access to ASD services, there were some study limitations. First, since our study was not a randomized controlled trial and did not include preintervention data, it is possible that toddlers in San Diego County have never experienced disparities in access to care due to factors specific to the county (e.g. the relative wealth of the county as a whole; the high level of available services). Relative to other parts of the country, San Diego is service-rich, with many early services supported by the SDRC. In fact, the most recent CDC report notes that the prevalence rates of autism in San Diego County are close to 4% (Maenner et al., 2021), suggesting that given the city’s strong service record, families do not leave San Diego County once they begin living in the county. Thus, economics, and state policies, potentially played a role in positive study outcomes. However, a large range of socioeconomic status levels, including those at poverty level, were captured across the areas screened (analyzed via parent-reported zip code; see Pierce et al., 2021), thus indicating that the program’s reach was not limited to a particular subset of wealth and may serve to address disparities in care across different HURM groups. Second, while San Diego has rich ethnic diversity with ~34% of children from Hispanic background, it is racially less diverse with only 5.5% Black children, making findings relating to race potentially less stable. Third, the exclusion of nondigital screens in this manuscript may have reduced disparity rates as families with lower eHealth literacy may have been excluded from analyses. However, it is unclear whether healthcare staff such as nurses and providers buffered this effect by assisting families in completing the screen should they have encountered difficulties. Fourth, one could speculate that there may be cultural biases within the Yes/No concerns checkbox the CSBS utilizes. However, previous literature has demonstrated that, aside from the social skills domain, there were no significant differences in the rate of endorsement of a range of developmental concerns (e.g. communication, problem behaviors, attention) across African American, White, or Hispanic parents of ASD children (Issarraras et al., 2019). Therefore, we hope that parents in our sample similarly felt comfortable answering the Yes/No question regardless of their background.

Importantly, our study revealed that there were no significant differences in the ages toddlers received an evaluation, entered ABA-based therapy, or the amount of ABA-based therapy received. The lack of significant differences within our variables of interest across ethnic and racial groups differs from overall findings in the Bilaver et al. (2021) study that demonstrated differences in rates of out-patient ASD services across Medicaid-enrolled groups. However, this may support the idea that the program’s benefits in ameliorating disparities do not drop off after the screen step in the screen-evaluate-treat chain and that having this chain was beneficial for getting HURM group members into services. These outcomes may also again be due to the fact that San Diego County, like other areas of California, contains state and federally funded nonprofit regional centers (e.g. SDRC) that increase the richness of resources within the area by providing and setting up services for those with developmental disabilities. Future research should examine if the implementation of a screen-evaluate-treat chain would be particularly useful to Medicaid-enrolled groups who may further struggle with receiving services. Finally, the disproportionate rate of evaluations compared to enrollment in treatment (i.e. higher number of evaluations in comparison with the number of treatment records based on records obtained or reported) may suggest that children may be receiving treatment through unidentified means or that there is a need in assisting families with access to services as, per UCSD ACE protocol, toddlers who received a diagnosis of any kind also received a referral for services unless they denied it or were already receiving the necessary support. It is also important to consider whether families were able to find support services they were comfortable with their child receiving and if this played a role in the number of reported treatment records.

Overall, the use of developmental screening programs such as *Get SET Early* that outlines a range of key steps in the screen-evaluate-treat chain could function to ensure equity in access to care, education, and resources for HURM populations. As all ethnic and racial backgrounds classified by the U.S. Census were represented in a comparable manner to that of San Diego County demographics (US Census Bureau, 2021), our study results suggest that implementation of the *Get SET Early* program is effective for a wide range of individuals and works to increase accessibility across the screen-evaluate-treat chain. In particular, the *Get SET Early* program has enabled screening and referrals for HURM communities within San Diego County that may have had difficulties in accessing resources and care. This may function to support equity in access to care for HURM communities—a positive trend observed in other programs as well (e.g. Feinberg et al., 2021; Zuckerman et al., 2021).

## Supplemental Material

sj-docx-1-aut-10.1177_13623613221147416 – Supplemental material for Examination of the impact of the Get SET Early program on equitable access to care within the screen-evaluate-treat chain in toddlers with autism spectrum disorder Click here for additional data file. Supplemental material, sj-docx-1-aut-10.1177_13623613221147416 for Examination of the impact of the Get SET Early program on equitable access to care within the screen-evaluate-treat chain in toddlers with autism spectrum disorder by Christie Pham, Elizabeth C Bacon, Andrea Grzybowski, Cynthia Carter-Barnes, Steven Arias, Ronghui Xu, Linda Lopez, Eric Courchesne and Karen Pierce in Autism
