# Supplementary material for: Examination of the impact of the Get SET Early program on equitable access to care within the screen-evaluate-treat chain in toddlers with autism spectrum disorder
Source: Autism. 2023 Jan 11;27(6):1790–802. doi: 10.1177/13623613221147416 (PMC10333446; doi:10.1177/13623613221147416)
Supplement: sj-docx-1-aut-10.1177_13623613221147416 – Supplemental material for Examination of the impact of the Get SET Early program on equitable access to care within the screen-evaluate-treat chain in toddlers with autism spectrum disorder [file sj-docx-1-aut-10.1177_13623613221147416.docx]

Supplemental Table 1 – *Get SET Early* Ethnic and Racial Engagement: Digital vs. Paper

Percentage of ethnic and racial indication on digital versus paper overall CSBS screens and Spanish screens.

Spanish eCSBS Comparisons:

- Hispanic eCSBS vs. Spanish eCSBS
  - Average screen age: Hispanic eCSBS = 16.98; Spanish eCSBS = 16.81
  - Screen outcome: *X*^2^ = 11.42, *p* = .001
  - Parent concern (% concern that fell into the range of concern): *X*^2^ = 1.50, *p* = .22
  - Referred (% referred that fell into the range of concern): *X*^2^ = 14.80, *p* < .001
- Not Hispanic eCSBS vs. Spanish eCSBS
  - Average screen age: Not Hispanic eCSBS = 17.44; Spanish eCSBS = 16.81
  - Screen outcome: *X*^2^ = 58.29, *p* < .001
  - Parent concern (% concern that fell into the range of concern): *X*^2^ = 2.01, *p* = .16
  - Referred (% referred that fell into the range of concern): *X*^2^ = 29.23, *p* < .001
- English eCSBS vs. Spanish eCSBS
  - Average screen age: English eCSBS = 17.21; Spanish eCSBS = 16.81
  - Screen outcome: *X*^2^ = 30.85, *p* < .001
  - Parent concern (% concern that fell into the range of concern): *X*^2^ = 1.88, *p* = .17
  - Referred (% referred that fell into the range of concern): *X*^2^ = 23.40, *p* < .001

Supplemental Table 2 – *Get SET Early* Screen outcome comparison

Screen outcomes for overall digital, paper, and combined Spanish screens. Average overall screen age: Digital = 17.21; Paper = 17.11; Spanish = 17.57. *Note: The number of Spanish screens in the screen outcome categories is less than the number who indicated ethnic/racial group as some screens were deemed invalid.*
